# Multi-Scale Attention Convolutional Network for Masson Stained Bile Duct Segmentation from Liver Pathology Images

**DOI:** 10.3390/s22072679

**Published:** 2022-03-31

**Authors:** Chun-Han Su, Pau-Choo Chung, Sheng-Fung Lin, Hung-Wen Tsai, Tsung-Lung Yang, Yu-Chieh Su

**Affiliations:** 1 Institute of Computer and Communication Engineering, National Cheng Kung University, Tainan City 701, Taiwan; h821231@gmail.com (C.-H.S.); pcchung@ee.ncku.edu.tw (P.-C.C.); 2 Division of Hematology and Oncology, Department of Internal Medicine, E-Da Hospital, Kaohsiung 824, Taiwan; shlintw@yahoo.com.tw; 3 Department of Pathology, National Cheng Kung University Hospital, Tainan City 704, Taiwan; hungwen@mail.ncku.edu.tw; 4 Kaohsiung Veterans General Hospital, Kaohsiung 813414, Taiwan; tlyang.dicom@gmail.com; 5 School of Medicine, I-Shou University, Kaohsiung 824, Taiwan

**Keywords:** semantic segmentation, attention, multi-magnification inputs, liver pathology, bile duct

## Abstract

In clinical practice, the Ishak Score system would be adopted to perform the evaluation of the grading and staging of hepatitis according to whether portal areas have fibrous expansion, bridging with other portal areas, or bridging with central veins. Based on these staging criteria, it is necessary to identify portal areas and central veins when performing the Ishak Score staging. The bile ducts have variant types and are very difficult to be detected under a single magnification, hence pathologists must observe bile ducts at different magnifications to obtain sufficient information. This pathologic examinations in routine clinical practice, however, would result in the labor intensive and expensive examination process. Therefore, the automatic quantitative analysis for pathologic examinations has had an increased demand and attracted significant attention recently. A multi-scale inputs of attention convolutional network is proposed in this study to simulate pathologists’ examination procedure for observing bile ducts under different magnifications in liver biopsy. The proposed multi-scale attention network integrates cell-level information and adjacent structural feature information for bile duct segmentation. In addition, the attention mechanism of proposed model enables the network to focus the segmentation task on the input of high magnification, reducing the influence from low magnification input, but still helps to provide wider field of surrounding information. In comparison with existing models, including FCN, U-Net, SegNet, DeepLabv3 and DeepLabv3-plus, the experimental results demonstrated that the proposed model improved the segmentation performance on Masson bile duct segmentation task with 72.5% IOU and 84.1% F1-score.

## 1. Introduction

According to the Global Hepatitis Report, viral hepatitis led to 1.34 million deaths in 2015 [1]. The WHO have also estimated that 788,000 people die from liver cancer per year, and viral hepatitis (such as hepatitis B and C) is the primary cause leading to hepatocellular carcinoma and cirrhosis. Statistics showed that the prevalence rate of liver disease in Asian had a higher proportion than all the other region [2,3].

In clinical diagnosis, liver disease is generally performed by liver biopsy, since hepatology images can provide information at the cellular level. Generally, the grading and staging of liver disease are evaluated by the Ishak Score system [4]. When scoring a liver biopsy, the pathologists have to observe the characteristics of the entire liver biopsy under a microscope or a digital image. The staging criteria of the Ishak Score include periportal or periseptal interface hepatitis, confluent necrosis, portal inflammation, focal lytic necrosis, apoptosis, focal inflammation, and architectural changes such as fibrosis or cirrhosis. Different standards require pathologists to diagnose in different magnifications to obtain an overall view information or a detailed cellular information. For instance, the fibrosis staging is assigned from 0 to 6, corresponding from normal to cirrhosis, and is observed using Masson stained liver biopsy (see Table 1). The staging is based on the findings that whether portal areas have fibrous expansion, bridging with other portal areas, or bridging with central veins, or even cirrhosis.

**Figure 1:**
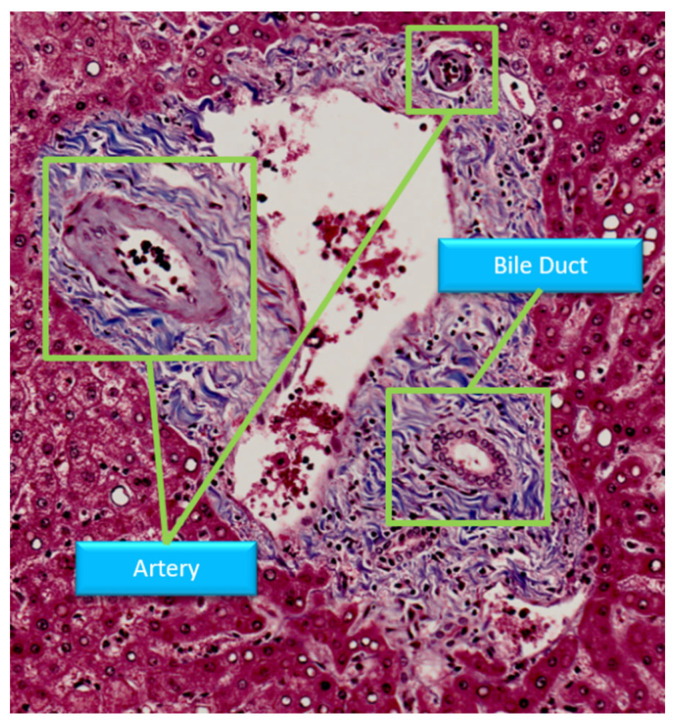
Structure of portal area in Masson stain.

According to the above application situation, in order to identify the fibrosis staging, it is necessary to first distinguish the central veins between portal areas. Generally, portal areas and central veins have different characteristics, and the most important is that bile ducts and arteries only appear in portal areas, as shown in Figure 1. Therefore, the findings of bile ducts and arteries in portal areas could solve the above distinction problem. Clinically, the bile ducts are considered as one of the criteria to identify portal areas. In addition, the detection of bile duct can also help diagnose bile duct cancer or vanishing bile duct syndrome. Therefore, in this study, a bile duct segmentation method is proposed to assist doctors and pathologists not only in staging the fibrosis score, but also in finding other bile duct diseases.

Automatic bile duct segmentation in liver pathology images, however, is challenging. First of all, since the color of the stained tissue gradually fades over time, and the staining protocols from different hospitals do not follow the same criteria, it is difficult to control the quality of the whole slide image (WSI) from liver biopsy. Secondly, even from the same liver biopsy image, different scanners can reveal color differences. The blurred area caused by the scanners and the fold region caused by the sectioning procedure also affect the display of the WSI. Most important of all, the bile ducts in different liver biopsies show variant types. The bile ducts from fibrous expansion, for instance, are thinner than normal bile ducts. The area and the number of bile ducts also differ in different portal areas. A larger portal area normally exists bile ducts clustering, or a huge bile duct with small ductules neighboring. Therefore, these factors increase the difficulty of automatic segmentation.

Accordingly, a multi-scale convolutional network architecture for the automatic segmentation of bile ducts in Masson stained from liver pathology images was proposed in this study. Three main concepts have been developed in the proposed model. First, the proposed method obtains features from both high-magnification and low-magnification patches in an attention method. The high-magnification view provides the microscopic feature but cannot provide enough macroscopic information, while low-magnification provides a larger view to solve the ambiguous features but loses the microscopic information. Hence, the multi-scale attention network solves the above problem by integrating low-magnification features and high-magnification features. Secondly, the integration is processed in an attention-grabbing way in order to avoid information received from low-magnification overly affecting the final segmentation result. Since the high-magnification patch is the main target to be segmented, the low-magnification features should be an assistant for the segmentation task. The patches extract from different magnifications are all generated in the same size, and the higher magnification patches are located in the central region of the lower magnification patches. Finally, the decoder structure in proposed model is to recover the lost information from low-level feature maps in encoder layer.

In order to verify the segmentation performance of the above design concepts in proposed model, two types of experiments were conducted in this study, including segmentation task compared with some existing models and the comparison of internal structural differences in proposed model. The experimental results demonstrated that the proposed model could improve the segmentation performance based on the above design concepts. From the measurement values and visual segmentation results, the contributions of the proposed model on bile duct segmentation from liver WSI would be addressed as follows:

- Both cell-level and adjacent structural feature information are integrated to simulate pathologists’ examination procedure for observing bile ducts under different magnifications in liver WSI;
- The attention method in proposed model would bring low-magnification input as an assistant help, enhancing the feature maps from high-magnification;
- The decoder structure of proposed model could recover the lost information from low-level feature maps in encoder layer, resulting in the obviously smooth segmentation boundaries;
- In clinical practice, the automatic analysis for pathologic examinations would contribute to the evaluation of the grading and staging of hepatitis based on Ishak Score system.

## 2. Related Works

Although there is still no research on the issue of automatic bile duct segmentation from liver pathology images, the segmentation and classification tasks focus on the medical filed have been increasing year by year, showing the appearance of novel methods in recent years significantly help in medical applications. Convolutional neural network is the main reason conduces to the development of image processing field. AlexNet [5], a classical convolutional neural network proposed by Krizhevsky et al., reached a new achievement in the ImageNet Large Scale Visual Recognition Challenge (LSVRC) in 2012, and is therefore leading to a great interest in convolutional networks among researchers. Compared to earlier image processing methods, convolutional neural networks have been improved more effectively, and therefore be applied in a large number of different fields including image classification [5,6], semantic segmentation [7], object detection [8], object generation [9], human pose estimation [10], diagnosis of diseases using EEG signals [11,12], and also an amount of applications in medical field.

### 2.1. Semantic Segmentation

The fully convolutional network (FCN) [7] is the first convolutional neural network structure applied to the semantic segmentation task and reached outstanding achievement. FCN provides an end-to-end training and pixel-wise predictions. Since the fully connected layer disrupt spatial information, the FCN architecture replaces all the fully connected layer by convolutional layer. The integration between different layers also helps segment the boundaries sharply. The FCN establish the foundation of semantic segmentation in deep learning. Accordingly, the U-net [13] architecture was proposed with a symmetric U-shaped encoder-decoder structure. In decoder, U-Net concatenates the layers from encoder at the same spatial resolution to recover lost information caused by pooling or convolution with stride 2, and gradually upsampled layer by layer to obtain finer boundaries. SegNet [14] also adopted a symmetric encoder-decoder structure similar to U-Net. In the encoder structure, SegNet stores the indices of the largest values in the max pooling layer. Then in the decoder, gradually upsample the layer and fills the value from the indices stored from corresponding positions in encoder. In recent years, DeepLab series [15,16,17,18] have been the standard for semantic segmentation network. The atrous spatial pyramid pooling (ASPP) is proposed to obtain the features at different receptive field to handle the problem of segmenting objects at multiple scales, and thus be widely applied in other segmentation networks. The comparison of above related works and proposed model is highlighted in Table 2.

### 2.2. Medical Image Task

In recent years, automatic analysis and diagnosis has attracted great interest in the medical field [19,20,21,22,23,24,25,26,27]. Due to the significant success in convolutional networks, deep learning has also been applied to medical images. Wang et al. [28] proposed an approach to combining both CNN and handcrafted features for mitosis detection, the dimensionality of handcrafted features was reduced with principal component analysis (PCA), and the features from two methods were classified by random forest classifiers. In lung cancer analysis, Cui et al. [29] applied the U-Net architecture for cancer cell detection, and an architecture based on VGG-Net with global average pooling to classify cells into different risk groups. In glioma research, Kurc et al. [30] applied a series of deep learning networks from digital pathology, the Mask-RCNN network with non-maximum suppression was adopted to nuclei segmentation in brain tissue images. Furthermore, the classification of brain cancer cases is applied in combining the predictions of both radiology image and pathology image, where a 3D CNN network is adopted as the radiology classification model and a DenseNet network pretrained on ImageNet is adopted as the histopathology classification model.

The multi-scale networks have also been applied in medical challenging tasks, since medical images is hard to predict in a single magnification. The information of different magnifications helps networks to obtain both local microscopic information and larger macroscopic information, and further help improve the prediction. In Liu et al. [31], a multi-scale approach applied with 40× magnification patches and lower magnification patches is proposed to classify breast cancer patches. Both inputs adopted Inception-v3 as the feature extraction model, and a fully connected layer is applied after two feature maps to combine features from different magnification information. Song et al. [32] also adopted a multi-scale approach to accurately segment cervical cytoplasm and nuclei. The multi-scale convolutional network is composed of multistage trainable architecture and each stage includes convolution layer, nonlinearity layer, and feature pooling layer. The proposed network is explored to extract scale invariant features, and segment regions centered at each pixel. Huang et al., and Sayıcı et al., both applied multi-scale magnification network to solve hepatocellular carcinoma classification task in H&E stained [33,34]. Huang et al., proposed a central region crop method to solve the alignment problem of local information in two different inputs to improve the performance, whereas Sayıcı et al., proposed an attention method to reweight the influences from different magnifications with softmax.

In this paper, a multi-scale input concept is adopted to obtain different fields of view information. The proposed multi-scale attention network integrates cell-level information and adjacent structural feature information for bile duct segmentation. In addition, the attention mechanism enables the network to focus the segmentation task on the input of high magnification, reducing the influence from low magnification input, but still helps to provide wider field of surrounding information. Our experimental result demonstrated that the multi-scale patches could improve the segmentation performance on Masson bile duct segmentation task, and our approach would achieve better performance of IOU and F1-score compared to other networks.

## 3. Materials and Methods

### 3.1. Multi-Magnification Patches

Clinically, pathologists and doctors examine the whole slide image on different magnifications to provide more spatial information, such as obtaining local information around the target object from high-magnification, or obtaining a global view of the entire tissue from low-magnification. Therefore, our study follows the same approach to feed images in multi-magnification, helping the model accurately segment bile ducts from additional local information.

**Figure 2:**
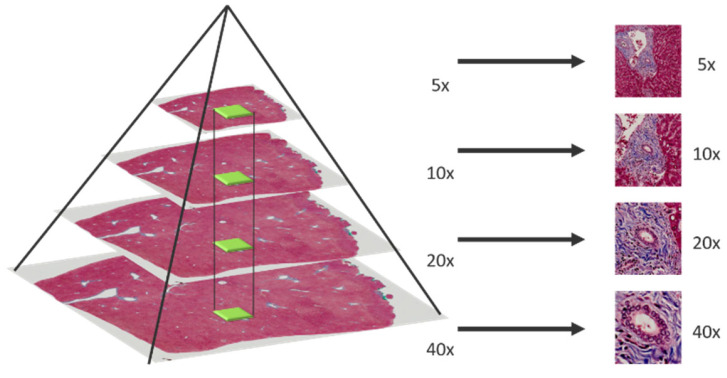
Multi-magnification patches generated by sliding window.

**Figure 3:**
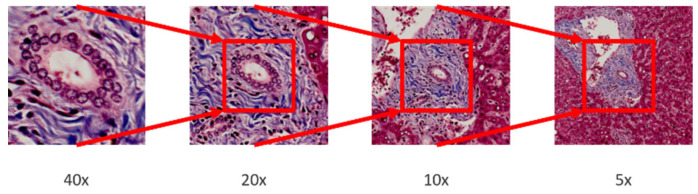
Patches from different magnifications. The higher magnification patch is located in the central region of the lower magnification patch.

In multi-magnification patches generation, an image pyramid building by applying bilinear interpolation is adopted to down sample the whole slide images (WSI) layer by layer from the highest magnification. After that, a sliding window fixed with size 256 × 256 moves from the whole slide image in different pyramid level to generate multi-magnification patches. Moreover, each patch generated in different pyramid level should center at the same location, higher magnification patches will locate in the central region of lower magnification patches. The process of multi-magnification patches generation is shown in Figure 2 and Figure 3.

While high-magnification patches provide detailed information from the object, low-magnification patches can obtain local information around the target to assist in the bile duct segmentation. However, the choosing of the two magnifications is important. Since bile ducts often be visible in a high-magnification level, a too much low-magnification input with useless background information will mislead the model to a wrong prediction. Therefore, the selection of input magnification has a significant influence on the model’s ability. In order to avoid the loss from resized patches in low-magnifications, in our experiment, we select the highest 40× magnification patches as the main input, meanwhile select 20× magnification patches as the secondary input to increase additional information.

### 3.2. Multi-Scale Attention Convolutional Network

Multi-scale Attention Convolutional Network (MACN) is an image segmentation architecture. The structure of the network is composed of three main concepts: (a) feature extraction from high-magnification and low-magnification images respectively by separate convolutional networks, (b) integration of the two magnification feature maps in an attention method, and (c) the decoder structure to recover the lost information from low-level feature maps in encoder layer.

According to the first concept of the present structure, the extraction applies two parallel convolutional neural networks, respectively. In this paper, the ResNet-101 [35] pretrained from ILSVRC-2012-CLS image classification dataset is utilized as the feature extraction network to reduce training time in our segmentation task. In order to further increase receptive field and obtain features from different scale of bile ducts, dense atrous spatial pyramid pooling block (DenseASPP) [36] is considered to be suitable framework, and hence concatenates after ResNet-101 block.

**Figure 4:**
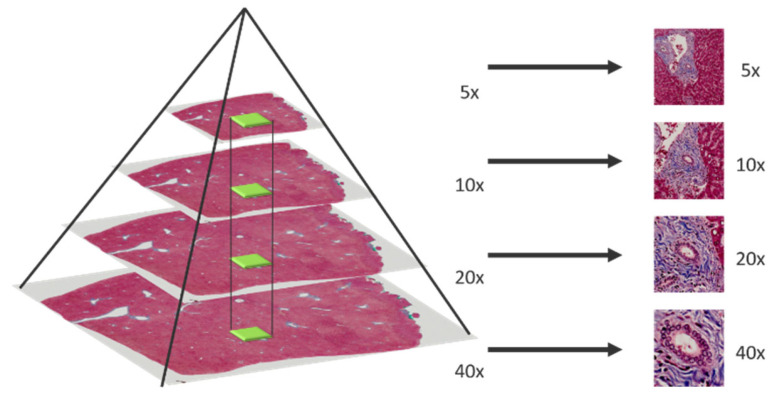
The central crop method to align different magnification feature maps.

In feature map integration, the alignment between two different magnification is important. Since the spatial information from different magnification feature map does not align on the same location, straightly doing convolution operation on multi-magnification feature maps can leads to the spatial information disruption. The central crop method is adopted to improve this problem, spatially-constrained integration phase to align the location of each feature map element between low magnification feature maps and high magnification feature maps, as shown in Figure 4.

**Figure 5:**
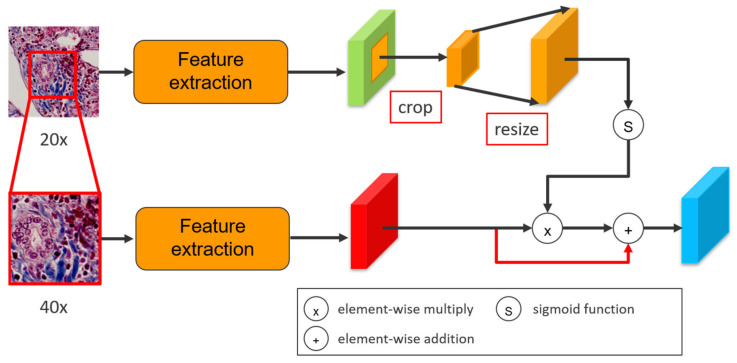
An attention method to enhance the high-magnification feature map.

**Figure 6:**
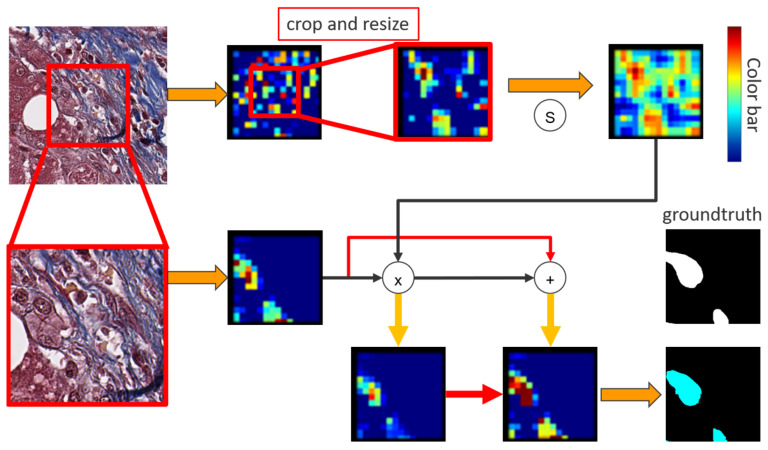
The attention method visualization.

Since the high-magnification input is the target prediction, an attention method is proposed to prompt low-magnification input as an assistant help, enhancing the feature maps from high-magnification. After central crop and bilinear resize, the low-magnification input pass through a sigmoid function to converge weights between [0, 1]. The useful information in the nodes of low-magnification feature maps will activate and approach 1, indicating the corresponding positions in low-magnification have significant local information. Afterwards, an element-wised multiplication in the two feature maps is generated to produce an attention feature map. In the attention map, the value of the nodes in low-magnification feature map approaching 1 preserve the value corresponding to the same location from high-magnification feature map. Concretely, the multiplication retains the value of the nodes when values in both two magnification patches are activated, indicating that the location in both two patches exists useful information. Furthermore, since the activated nodes from low-magnification feature map multiply with the nodes from high-magnification feature map that are not activated will still remain low value, the attention method can sufficiently control the influence from low-magnification input, and therefore can be considered as an enhancement. Lastly, an element-wised addition between attention feature map and high-magnification feature map is adopted to enforce the values of high-magnification feature map. The attention method can be defined as:

(1) Y(x)=(1+σ(L(x)) )∗H(x)

where *Y*(*x*) is the output, *σ* is a sigmoid function, *L*(*x*) and *H*(*x*) are low-magnification and high-magnification feature maps, respectively. Figure 5 and Figure 6 shows the whole process of the attention method.

In order to refine the segmentation results especially along object boundaries. The decoder structure similar to [13] and [18] is also applied. The decoder module that gradually recovers the spatial information obtains sharper boundaries. In detail, the features after attention method are bilinearly upsampled by a factor of 2 and then concatenated with the corresponding low-level features from the ResNet-101 network, then apply convolution layers to merge the features.

### 3.3. Focal Loss

In this study, we change the traditional loss method from cross-entropy to focal loss [37], which get a great success in object detection. The cross-entropy loss can be defined as:

(2) $$CE\left(p_{t}\right)=-ylog\left(p_{t}\right)$$

where $p_{t}$ is the probability predicted from model, and y is the ground truth.

Focal loss is extended from cross entropy to focus the loss calculation in challenging data, the equation can be defined as:

(3) $$FL\left(p_{t}\right)=-y{\left(1-p_{t}\right)}^{r}log\left(p_{t}\right)$$

where r is a hyperparameter default as 2.

The equation of focal loss will reduce the influence of easy prediction object. For instance, if a prediction of probability from model is 0.9 and the label is 1, it indicates the pixel is easy to predict, and thus decrease its influence on the model weights update, lowering 100× loss compared with cross-entropy (r = 2). The challenging task will significantly affect the loss. Focal loss adopted in medical images also have a big influence since medical images are various, so we changed the traditional cross-entropy method to focal loss to help correct prediction.

### 3.4. Basic Model Operations

In this section, the basic model operations such as activation function or batch normalization employed in our proposed models are described more specifically in the following section parts.

#### 3.4.1. Activation Function

The activation function is usually an abstraction concept inspired by biological neural network, representing the activation of cells. In artificial neural networks, we generally add activation functions to simulate the operation of biological neurons and generate non-linear transformations to increase model complexity. The Rectified Linear Unit (ReLU) [38] has been widely used as an activation function in neural network structure, providing the sparsity and preventing model from vanishing gradient problems compared to other activation functions, like sigmoid or hyperbolic tangent. The Rectified Linear Unit (ReLU) function can be defined as:

(4) ReLU(x)=max (0,x)

where x is the summation of weights from previous layers. In our model, we apply ReLU as the default activation function after the convolution layer and the fully connected layer.

The SoftMax function is a generalization of the logistic function that “squashes” the value of a vector into range [0, 1], and all the values add up to 1. The output of SoftMax function is used to represent a probability distribution in probability theory. That is to say, the output can be considered as the individual probability of different input values in the vector, making SoftMax function widely used in the final output layer of neural networks. The predicted probability of the *j*_th class from SoftMax function can be defined as:

(5) $$P\left(y=j|x\right)=\frac{e^{x^{T}}w_{j}}{\sum_{k=1}^{K}e^{x^{T}}w_{k}}$$

where *K* is the total number of the class, and x is a vector of output layer in neural network.

#### 3.4.2. Batch Normalization

Batch normalization [39] is a hidden layer feature normalization approach. The approach id applied to avoid covariate shift problem at the hidden layers. Covariate shift problem means that the distribution of the different data features has a large variance in the training phase. If the network goes deeper, the problem will be spread in the deep layers. The batch normalization forces the mean of the batch data features be 0 and the standard deviation of the batch data features be 1, and it makes the training more stable. The batch normalization equation $BN_{r,\beta }\left(x_{i}\right)$ can be defined as:

(6) $$\mu_{\beta }=\frac{1}{m}\sum_{i=1}^{m}x_{i}$$

(7) $$\sigma_{\beta }^{2}=\frac{1}{m}\sum_{i=1}^{m}{\left(x_{i\;}-\mu_{\beta }\right)}^{2}\;$$

(8) $${\hat{x}}_{l}=\frac{x_{i\;}-\mu_{\beta }\;}{\sqrt{\sigma_{\beta }^{2}}+\varepsilon }$$

(9) $$BN_{\gamma ,\;\;\beta }(x_{i})=\gamma {\hat{x}}_{l}+\beta$$

where m is batch size, $x_{i}$ is $i_{th}$ data in batch, $\mu_{\beta }$ is the mean of the batch, $\sigma_{\beta }^{2}$ is the standard deviation of the batch, ${\hat{x}}_{l}$ is the $i_{th}$ normalized value in the batch, *ε* is a small constant for numerical stability and the *γ* and *β* are trainable parameters for distribution scale and shift.

## 4. Results

### 4.1. Masson Stained Dataset

The dataset used in this study was obtained from the Department of Pathology, National Cheng Kung University Hospital approved by the Institutional Review Board and diagnosed by experienced pathologist. The scanners currently serving in the hospital is captured as a WSI at 40× magnification using an Aperio AT2–Digital Whole Slide Scanner (Leica Biosystems Imaging, Inc., Wetzlar, Hesse, Germany). In the semantic segmentation of the WSIs, the dataset contains 60 whole slide images from cases with diagnosis confirmed by specialist. The training set includes 32 cases and the testing set contains 28 cases. In the experiments, after pyramid patch generation, a total of 215,607 patches were generated with each patch resolution fixed at 256 × 256. However, most of these patches are segmented from hepatocyte areas, which might result in an imbalance for our training and thus influence the prediction capability. To reduce the proportion of hepatocyte patches in total dataset, patches that include fibrosis area, artery, vein and bile duct was retained. With this strategy, the total patches were reduced to 40,120 in the dataset and 23,822 and 17,298 patches were included as training and testing set, respectively. The bile duct pixels were labeled as foreground, and others as the background.

### 4.2. Evaluation

In the experiment, the proposed segmentation network was compared to recently segmentation networks. The experiment was designed to evaluate the performance of each segmentation network in the histopathological image dataset.

In this study, Precision (P), Recall (R), and F1-score were used to quantify the segmentation performance. The aim of the proposed method is to segment the bile duct from each patch. To this end, a pixel segmentation point is considered as true-positive (TP) if the point is located within bile duct. In contract, a pixel segmentation point is considered as false-positive (FP) if the point is in other areas such as fibrosis, artery, vein and hepatocyte. The TP, FP, TN and FN were formulated in Table 3.

According to the above definition, equations that include true-negative (TN) were excluded because most of the pixels are predicted as negative. Hence, the main criteria for evaluation are defined as follows:

(10) $$\mathrm{Precision}\;\left(P\right)=\frac{\mathrm{TP}}{\mathrm{TP}+\mathrm{FP}}$$

(11) $$\mathrm{Recall}\left(R\right)=\frac{\mathrm{TP}}{\mathrm{TP}+\mathrm{FN}}$$

(12) $$F1\;\mathrm{score}\;=\frac{2*R*P}{R+P}$$

(13) $$\mathrm{IOU}\;=\frac{\mathrm{GroundTruth}\;\cap \;\mathrm{DetectionResult}}{\mathrm{GroundTruth}\;\cup \;\mathrm{DetectionResult}}=\frac{\mathrm{TP}}{\mathrm{TP}+\mathrm{FP}+\mathrm{FN}}$$

Table 4 shows the performance between the proposed network and other segmentation networks. The performance of precision and F1-score and IOU in our proposed network are higher than others in testing cases, while recall value is similar to DeepLabv3 and DeepLabv3-plus. The results suggest that capability of these networks to identify bile duct is similar. However, our proposed network has an advantage of diminishing false positive predictions.

In addition to the above experiment of proposed and other segmentation networks, comparison of internal structural differences in proposed model was also conducted, including the performance between attention mechanism and concatenation, crop/without crop the central region in the feature map integration, integration the feature map after DenseASPP block or after ASPP block, model with/without decoder, and the performance of loss methods between cross-entropy and focal loss.

## 5. Discussion

### 5.1. Visual Experiment Results

**Figure 7:**
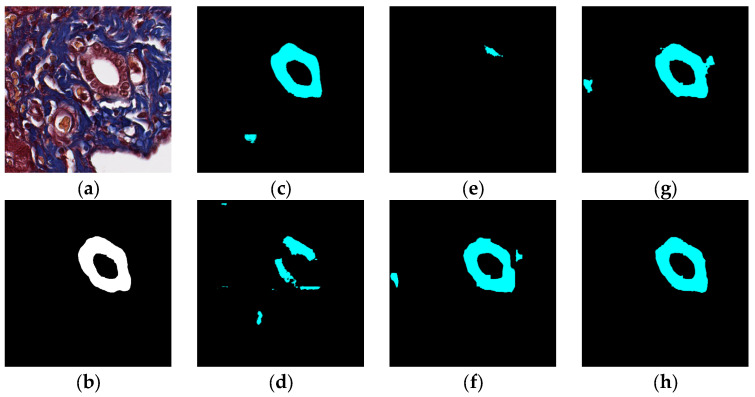
Segmentation results for clearly visible bile ducts: original (**a**), GT (**b**), FCN (**c**), SegNet (**d**), U-Net (**e**), Deeplabv3 (**f**), Deeplabv3-plus (**g**), and the proposed network (**h**).

**Figure 8:**
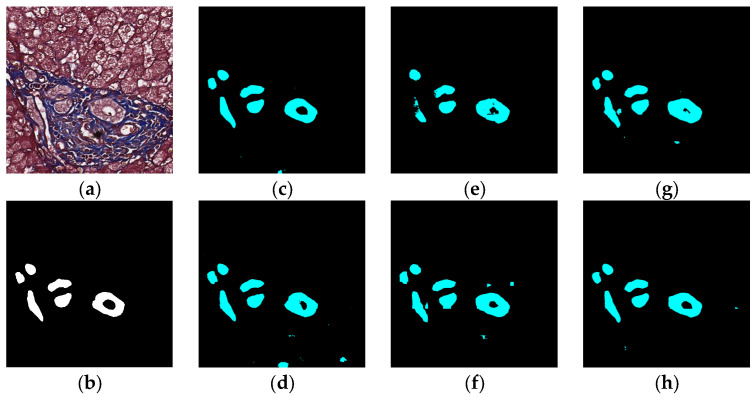
Segmentation results for small bile ducts: original (**a**), GT (**b**), FCN (**c**), SegNet (**d**), U-Net (**e**), Deeplabv3 (**f**), Deeplabv3-plus (**g**), and the proposed network (**h**).

**Figure 9:**
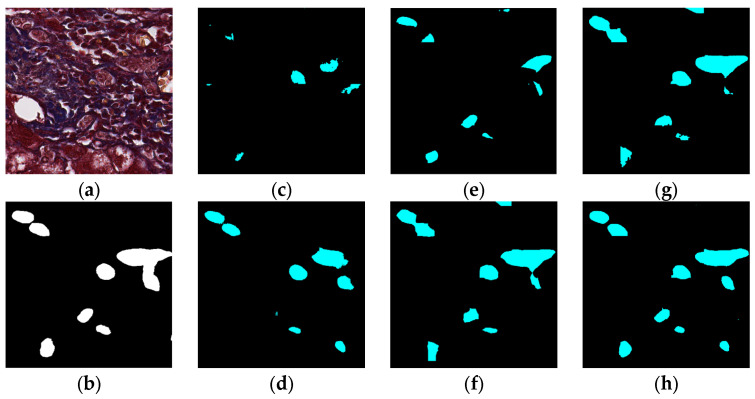
Segmentation results for small ductules stained similar to hepatocyte area: original (**a**), GT (**b**), FCN (**c**), SegNet (**d**), U-Net (**e**), Deeplabv3 (**f**), Deeplabv3-plus (**g**), and the proposed network (**h**).

**Figure 10:**
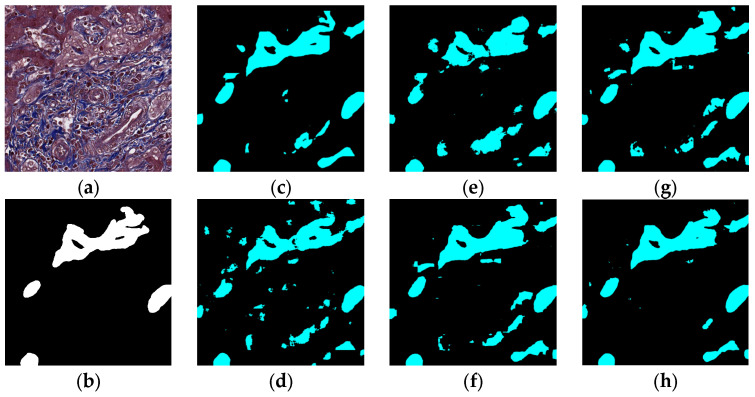
Segmentation results for the case in which cells in arteries are much similar to real bile ducts: original (**a**), GT (**b**), FCN (**c**), SegNet (**d**), U-Net (**e**), Deeplabv3 (**f**), Deeplabv3-plus (**g**), and the proposed network (**h**).

**Figure 11:**
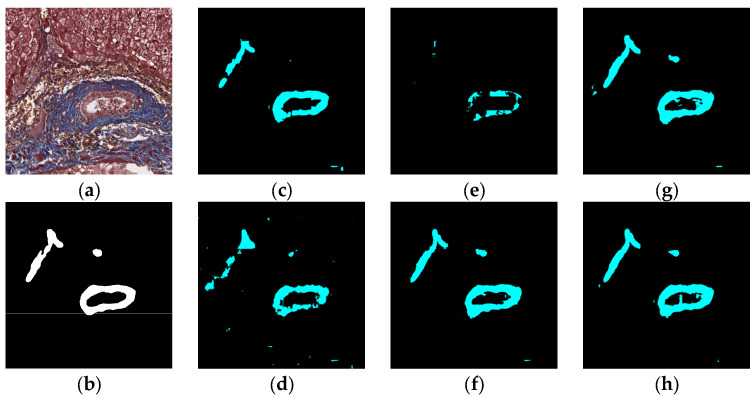
Segmentation results for big bile ducts and bile ductules near hepatocyte area: original (**a**), GT (**b**), FCN (**c**), SegNet (**d**), U-Net (**e**), Deeplabv3 (**f**), Deeplabv3-plus (**g**), and the proposed network (**h**).

**Figure 12:**
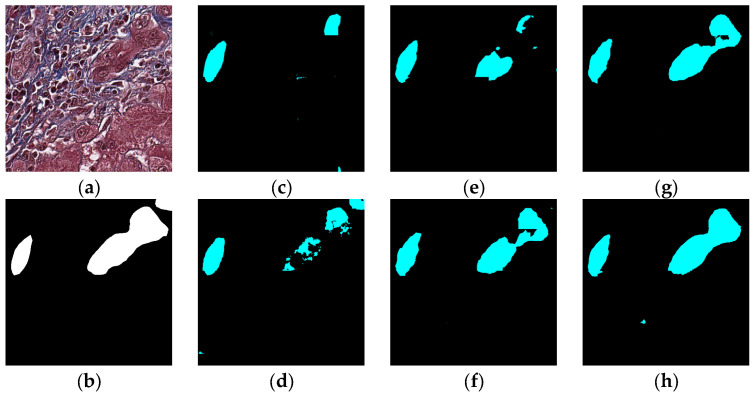
Segmentation results for the case in which the bile duct is between the hepatocyte area and the classic bile ducts: original (**a**), GT (**b**), FCN (**c**), SegNet (**d**), U-Net (**e**), Deeplabv3 (**f**), Deeplabv3-plus (**g**), and the proposed network (**h**).

The visual segmentation results of existing and proposed models are shown in Figure 7, Figure 8, Figure 9, Figure 10, Figure 11 and Figure 12. As shown in Figure 7, our proposed network correctly segmented the clearly visible bile ducts and significantly reduced incorrect predictions in hepatocyte area in comparison with other networks. As for the case with small bile ductules, the visual segmentation results in Figure 8 illustrated that the proposed model could accurately segment the small bile ductules with reduced incorrect predictions in hepatocyte area. The importance of decoder structure can be illustrated in Figure 9 in which the object boundaries are smoother in our network. In addition, the proposed network also demonstrated the ability to find small ductules stained similar to hepatocyte area in Figure 9. Figure 10 demonstrated a difficult case of segmentation task in which cells in arteries are much similar to real bile ducts. From the visual segmentation results in Figure 10, all other compared models gave erroneous predictions on these arteries, but our network had fewer incorrect predictions than others. In Figure 11, compared to other networks, our network segmented the big bile duct and bile ductules near hepatocyte area more completely. Figure 12 shows a special case of bile duct. The type of the bile duct in Figure 12 is between the hepatocyte area and the classic bile ducts, and thus is difficult to be segmented correctly. From the visual segmentation results in Figure 12, our model achieved a smoother segmentation result. Since the types of bile ducts and ductules vary between different cases, the segmentation results of FCN, U-Net, SegNet are fragmented and incomplete, while the DeepLab series and the proposed network perform more completely. Furthermore, the proposed network had a better ability to reduce segmentation errors in hepatocyte areas, arteries and fibrosis areas.

As for the comparison of internal structural differences in proposed model, Table 5 shows that in the model without crop, the local information of low magnification input will disrupt spatial information, resulting in increasing false positive predictions in wrong area and declining the precision value. Similarly, simply concatenating two different magnification would increase more unnecessary information from high magnification input. Hence, the model with attention method to focus the information on high magnification could significantly improve the performance, as shown in Table 6.

Table 7 shows the comparison between the performance of model with DenseASPP block and with ASPP block. The performance in both methods is similar, but the model with DenseASPP block would perform better owing to the increase of complexity and receptive field. Table 8 shows the performance between model with and without decoder. Although the advantage of decoder structure does not show a significant influence on the values of evaluation criterion compared to the structure without decoder, it does obviously smooth the segmentation boundaries that could be observed in the visible results.

Lastly, the performance comparison of focal loss with different gamma values is shown in Table 9. Since gamma value definitely decreases the total loss value, the higher gamma value will get lower performance in which total loss is too low to modify model weights.

### 5.2. Limitations of the Study in Digital Pathologyl Image Analysis

The clinical diagnosis of liver diseases is generally performed with liver biopsy since pathologists could examine the liver biopsy at different magnifications under microscope or digital image for more detailed and precise information at both structural and cellular levels. This labor intensive and expensive examination process, however, would waste lots of pathologists’ working time. In order to reduce the intensive labor costs and potential human diagnose error, automatic diagnosis and assistance for pathologists are extremely important. Thus, the proposed model could provide efficient and precise segmentation results for pathologists to distinguish bile ducts in WSI.

Owing to that the labeling job for digital pathological image is time consuming and the digital pathological image has features of huge file size (over 10 GB in average), it is quite difficult to obtain open dataset of digital pathological images labeled by pathologists. Thus, to obtain more precise segmentation results it would be necessary to increase the labeled cases and/or to perform post-processing mechanism to reduce the false-positive rate and keep the IoU score. In spite of these limitations of image segmentation task on digital pathological images, it would be great helpful assistance in clinical practice when adopting suitable and advanced AI model in digital pathology examinations to provide more efficient and precise segmentation results for pathologists.

## 6. Conclusions

This paper presents a multi-scale deep convolution neural network with integration of different magnification feature maps in an attention method. Three main concepts have been developed in our model: (a) feature extraction from high-magnification and low-magnification images respectively by separate convolutional networks, (b) integration of the two magnification feature maps in an attention method, and (c) the decoder structure to recover the lost information from low-level feature maps in encoder layer. As shown in our results, multi-scale deep attention neural network demonstrates the multi-scale patches that could improve the segmentation performance on Masson bile duct segmentation task. Furthermore, 72.5% IOU and 84.1% F1-score were achieved in our approach, better than other networks reported earlier.

Although the labeling job for digital pathological image is time consuming, we are still ongoing to increase the labeled case to improve the accuracy of the proposed model. In addition, a new post-processing mechanism would be tried to reduce the false-positive rate and keep the IoU score. The post-processing mechanism in development is to provide confirmed patches and candidate for doctors to quickly filter the candidate patches.

## Figures and Tables

**Table 1:** Ishak fibrosis staging.

Status	Score
No fibrosis	0
Fibrous expansion of some portal areas, with or without short fibrous septa	1
Fibrous expansion of most portal areas, with or without short fibrous septa	2
Fibrous expansion of most portal areas with occasional portal to portal (P-P) bridging	3
Fibrous expansion of portal areas with marked bridging; portal to portal (P-P) as well as portal to central (P-C)	4
Marked bridging (P-P and/or P-C) with occasional nodules (incomplete cirrhosis)	5
Cirrhosis probable or definite	6

**Table 2:** Comparison of related works and proposed model.

	Comparison	Strengths	Weaknesses
Model			
FCN		fast learning and inference	segmentation performance
U-net		finer segmentation boundary; training with very few images	high computational resources
SegNet		low computational memory	high computational time
DeepLabv3		augmenting ASPP for better performance	poor segmentation boundary
DeepLabv3-plus		finer segmentation boundary	high computational resources
proposed		finer segmentation boundary; high segmentation performance	high computational resources

**Table 3:** TP, FP, TN and FN performance metrics.

	Ground Truth	Positive	Negative
Prediction			
Positive		True positive (TP)	False positive (FP)
Negative		False negative (FN)	True negative (TN)

**Table 4:** Performance between semantic segmentation networks.

	Evaluation	Precision	Recall	F1-Score	IOU
Model					
FCN [7]		0.818	0.770	0.793	0.657
SegNet [14]		0.637	0.678	0.657	0.489
U-net [13]		0.590	0.723	0.651	0.482
DeepLabv3 [17]		0.769	0.857	0.810	0.681
DeepLabv3-plus [18]		0.775	0.855	0.813	0.685
Proposed		0.824	0.858	0.841	0.725

**Table 5:** Performance between model with crop and without crop.

	Evaluation	Precision	Recall	F1-Score	IOU
Method					
With crop (Proposed)		0.824	0.858	0.841	0.725
Without crop		0.760	0.866	0.810	0.680

**Table 6:** Performance between model with attention and with concatenation.

	Evaluation	Precision	Recall	F1-Score	IOU
Method					
Attention (Proposed)		0.824	0.858	0.841	0.725
Concatenation		0.772	0.850	0.809	0.680

**Table 7:** Performance between model with DenseASPP and with ASPP.

	Evaluation	Precision	Recall	F1-Score	IOU
Method					
DenseASPP (Proposed)		0.824	0.858	0.841	0.725
ASPP		0.838	0.838	0.838	0.722

**Table 8:** Performance between model with decoder and without decoder.

	Evaluation	Precision	Recall	F1-Score	IOU
Method					
With decoder (Proposed)		0.824	0.858	0.841	0.725
Without decoder		0.822	0.852	0.837	0.720

**Table 9:** Performance of focal loss between different gamma values.

	Evaluation	Precision	Recall	F1-Score	IOU
Method					
gamma=0 (Cross Entropy)		0.806	0.852	0.829	0.707
gamma = 1 (Proposed)		0.824	0.858	0.841	0.725
gamma = 2		0.823	0.840	0.832	0.712
gamma = 3		0.807	0.837	0.822	0.698
gamma = 4		0.775	0.839	0.806	0.675

## Data Availability

Not applicable.
